# The reliability, validity, and preliminary responsiveness of the Eye Allergy Patient Impact Questionnaire (EAPIQ)

**DOI:** 10.1186/1477-7525-3-67

**Published:** 2005-10-31

**Authors:** Michael Alexander, William Berger, Patricia Buchholz, John Walt, Caroline Burk, Jeff Lee, Rob Arbuckle, Linda Abetz

**Affiliations:** 1 Niagara Clinical Research, 5673 North Street, Niagara Falls, Ont L2G1J4, Canada; 2 Southern California Research, 27800 Medical Center Road, Suite 240, Mission Viejo, CA 92691, USA; 3 Allergan, Inc., Ettlingen GmbH, Pforzheimer Str. 160, Ettlingen 76275, Germany; 4 Allergan, Inc., 2525 Dupont Drive, Irvine, CA 92612, USA; 5 CT Burk, Inc., 1337 Cerritos Drive, Laguna Beach, CA 92651, USA; 6 Mapi Values Ltd, Adelphi Mill, Grimshaw Lane, Bollington, Macclesfield, Cheshire SK10 5JB, UK

**Keywords:** Patient functioning, ocular allergy, psychometric validation, EAPIQ, patient reported outcomes

## Abstract

**Background:**

The Eye Allergy Patient Impact Questionnaire (EAPIQ) was developed based on a pilot study conducted in the US and focus groups with eye allergy sufferers in Europe. The purpose of this study was to present the results of the psychometric validation of the EAPIQ.

**Methods:**

One hundred forty six patients from two allergy clinics completed the EAPIQ twice over a two-week period during the fall and winter allergy seasons, along with concurrent measures of health status, work productivity, and utility. Construct validity, reliability (internal consistency and test-retest), concurrent, known-group, and clinical validities, and responsiveness of the EAPIQ were assessed. Known-group validity was assessed by comparing EAPIQ scale scores between patients grouped according to their self-rating of ocular allergy severity (no symptoms, very mild, mild, moderate, severe, very severe). Clinical validity was assessed by assessing differences in EAPIQ scores between groups of patients rated by their clinician as non-symptomatic, mild, moderate, and severe.

**Results and Discussion:**

Results from the validation study suggested the deletion of 14 of 43 items (including embedded questions) that required patients to complete the percentage of time they were troubled by something (daily activity limitations/emotional troubles). These items yielded a significant amount of missing or inconsistent data (50%). The resulting factor analysis suggested four domains: symptoms, daily life impact, psychosocial impact, and treatment satisfaction. When included as separate scales, the symptom-bother and symptom-frequency scales were highly correlated (> 0.9). As a consequence, and due to superior discriminative validity, the symptom bother and frequency items were summed. All items met the tests for item convergent validity (item-scale correlation = 0.4). The success rate for item discriminant validity testing was 97% (item-scale correlation greater with own scale than with any other). The criterion for internal consistency reliability (alpha coefficient ≥ 0.70) was met for all EAPIQ scales (range 0.89–0.93), as was the criterion for test-retest reliability (intraclass correlation [ICC] ≥ 0.70). Largely moderate correlations between the scales of the EAPIQ and the mini Rhinoconjunctivitis Quality of Life Questionnaire (miniRQLQ) and low correlations with the Health Utilities Index 2/3 (HUI2/3) were indicative of satisfactory concurrent validity. The EAPIQ symptoms, Daily Life Impact, and Psychosocial Impact scales were able to distinguish between patients differing in eye allergy symptom severity, as rated by patients and clinicians, providing evidence of satisfactory known-group and clinical validities, respectively. Preliminary analyses indicated the EAPIQ Symptoms, Daily Life Impact, and Psychosocial Impact scales to be responsive to changes in eye allergies.

**Conclusion:**

Following item reduction, construct validity, reliability, concurrent validity, known-group validity, and preliminary responsiveness were satisfactory for the EAPIQ in this population of ocular allergy patients.

## Background

The term ocular allergy is used to describe a number of distinct disease entities, ranging from allergic conjunctivitis, a relatively mild condition, to keratoconjunctivitis, a sight-threatening condition affecting the cornea [1]. All could be described as atopic conditions affecting the conjunctiva and the surrounding structures of the eye, including the eyelids. Underlying immune mechanisms responsible have not been clarified, but it is believed that IgE related mast cell and eosinophil mediated inflammation leads to the release of mast cell mediators and toxic eosinophil granule proteins and enzymes. Ocular allergy affects approximately 15% of the world population, and its incidence is increasing in industrialised countries [2]. Elsewhere it has been reported that approximately 20% of the population in temperate climates suffer from allergic rhinoconjunctivitis [3]. Patients suffering from ocular allergy might experience such symptoms as red, itchy, burning, swollen or dry eyes in differing degrees of severity and duration. Some patients might only be affected for a few weeks, while others may experience symptoms continuously throughout the year. Thus, ocular allergy potentially affects patients in their daily life activities, thereby impacting their health-related quality of life (HRQoL). In particular, people suffering from ocular allergy may be limited in performing daily activities such as reading, computer work, and going outside.

In order to treat patients effectively it is necessary to know which treatments work best and which treatments patients prefer to use. Patient reported outcomes instruments can be used to determine which drugs have the greatest effect on patient reported HRQoL, treatment satisfaction, and work productivity. Given the plethora of drugs on the market, patient reported outcomes data can provide patients and clinicians guidance as to which treatments are most beneficial for ocular allergy patients.

The EAPIQ (Appendix [see Additional file 1]), an ocular allergy-specific questionnaire, was recently developed to evaluate the impact of eye allergies on patient functioning and daily activities, and to assess patient satisfaction with treatment, for use in clinical trials. In addition, three questionnaires measuring the HRQoL of patients with ocular allergy have been identified in the literature. They are as follows: the Rhinoconjunctivitis Quality of Life Questionnaire (RQLQ) with standardised activities, the miniRQLQ and the Allergic Conjunctivitis Quality of Life Questionnaire. Previous versions of the EAPIQ have been used in studies in Europe and US, and results have been presented as posters [4,5]. The objective of the present study was to further validate the questionnaire by investigating the psychometric properties of the EAPIQ in a sample of patients with ocular allergies, in US and Canada.

## Methods

### Subjects and study design

This was an observational validation study involving patients with ocular allergy (data collected between October 2002 and March 2003). There were 146 ocular allergy patients in two allergy clinics in US and Canada. All 146 patients were administered the EAPIQ, and the two concurrent measures at baseline seven to ten days later, 79 of these patients were administered the EAPIQ a second time in addition to the Health Change questionnaire (for the assessment of test retest reliability).

The patients were stratified by the clinicians into four groups based on the severity of their symptoms based on their clinic experience: 'no current symptoms' (n = 34), 'mild symptoms' (n = 40), 'moderate symptoms' (n = 42), and 'severe symptoms' (n = 30).

### Measures

The following measures were administered during the study:

#### EAPIQ (Appendix [see Additional file 1])

A patient perspective questionnaire consisting of 49 items developed to measure ocular allergy symptoms and their impact on HRQoL, work productivity and treatment satisfaction. The EAPIQ was developed at Allergan from ocular allergy related questions derived from the mini Rhinoconjunctivitis QOL Questionnaire (mini RQLQ, Juniper et al.2000). Its structure, format, and layout was patterned after questions from the Ocular Surface Disease Index (OSDI, Walt et al.). The EAPIQ was presented to four patient focus groups (n = 10 in each group) in 2001 in UK, France, Italy and Sweden where language specific questionnaires (controlled by forward-backward translations) were generated for non- English groups. Patients were asked to comment on layout, structure, and clarity of questions. Based on these focus groups, the EAPIQ was restructured and questions were rephrased to be more patient friendly and concise. Further validation of the EAPIQ was conducted using the revised questionnaire at two allergy clinics in the US and Canada (146 patients).

Of the 49 original items in the questionnaire, the 43 items assessing symptoms (1–12), the impact of symptoms on HRQoL (items 18–31) and treatment satisfaction (items 32–34) were included in the item reduction and psychometric validation analyses. Six items assessing healthcare resource utilisation (item 13), work status (items 14 and 15), work productivity (items 16 and 17), and activity limitations (item 35) were not assessed in the analyses described because they require categorical and non-Likert type responses. Scores for the EAPIQ scales are transformed to give a minimum score of 0 and a maximum score of 100. Higher scores indicate a greater impact on health due to worse symptoms.

#### Mini Rhinoconjunctivitis Quality of Life Questionnaire (miniRQLQ)

A 14-item self-administered questionnaire developed by Elizabeth Juniper (MCSP, MSc) to measure the problems that adults with rhinoconjunctivitis experience in their day-to-day lives [6]. The miniRQLQ has five domains: activity limitations, practical problems, nose symptoms, eye symptoms and non-nose/eye symptoms.

#### Health Utilities Index (HUI2/3)

A health status and preference-based health-related quality of life measure suitable for use in clinical and population studies [7]. This 17 item self-administered questionnaire consists of seven attributes: sensation (vision, hearing, speech), mobility, emotion, cognition, self-care, pain, and fertility. The fertility dimension was excluded.

#### Health Status Change Questionnaire-Short Form

Administered at follow up, this questionnaire use six questions to assess the extent of any health change in the patient 7–10 days after the baseline assessment. Responses were used to categorise patients' health as 'better', 'stable', or 'worse' in order to assess the responsiveness of the EAPIQ.

### Analyses

Exploratory Factor Analysis (principal components analysis) with the number of factors left free was performed to categorise each item to its respective domains. The methodology used thereafter utilised the information gained from the factor analysis. The number of factors selected was determined by the number of factors that provided more than a 0.5 step in eigen value, ± 2 factors. Consideration was also made of the number of factors with eigen values > 1.0. Items were considered for deletion if they loaded on two or more factors, or had a correlation of less than 0.40 with their own factor, or had a high (> 0.70) floor or ceiling effect (based on item-descriptive statistics). However, if items were found to have substantial face or content validity they may still be retained, regardless of the factor analysis results.

The EAPIQ was then assessed for the following psychometric properties: item convergent validity [8,9], item discriminant validity [10], internal consistency reliability [11,12], test retest reliability [13], floor and ceiling effects, scale-scale correlations, concurrent validity [14], known-group validity, clinical validity and responsiveness [14], all defined in Table 1.

**Table 1:** Purpose of psychometric tests

**Property**	**Purpose**
Item convergent validity	To assess an item's correlation with its own hypothesized sub-scale score (satisfied if correlation achieved is ≥ 0.40)
Item discriminant validity	To assess whether an item considered in isolation has a higher correlation with its hypothesized scale than with other scales in the questionnaire
Internal consistency reliability	To evaluate the extent to which individual items of the instrument are consistent to one another and reflect an underlying scheme or construct (satisfied if Cronbach's alpha coefficient = 0.70 is achieved)
Test-retest reliability	Assesses the extent to which the measure yields the same results in repeated applications in an unchanged population. The intra-class correlation coefficient (ICC) was used as a measurement of test-retest reliability, and was assessed in patients who reported their health status to be stable between baseline (week 0) and study end (7 to 10 days later) (satisfied if an ICC coefficient = 0.70 is achieved)
Floor and ceiling effects	Refer to a high percentage of patients scoring the lowest score possible and a high percentage of patients achieving the highest score possible, respectively. High baseline floor or ceiling effects are indicative of a scale that is limited in its responsiveness to clinical change. Minimal floor and ceiling effects are therefore recommended. For the EAPIQ scales a percentage of 20% at floor or at ceiling was considered a significant effect
Scale-scale correlations	To determine whether the concepts measured in the individual scales (domains) of the EAPIQ were distinct and that none of the domains were redundant
Concurrent validity	Concurrent validity was supported if the EAPIQ sub-scales were substantially correlated (≥ 0.40), with miniRQLQ sub-scales measuring similar concepts. Conversely, sub-scales measuring unrelated concepts should be poorly correlated. As a generic measure of health status the HUI2/3 was expected to be less strongly correlated with the EAPIQ scales
Known-group validity	Differences in EAPIQ scores were expected among groups of patients known to differ in their patient-evaluated health status
Clinical validity	Clinical validity assesses the ability of scores to discriminate among groups of patients defined according to clinical severity. Patients who have a good clinical status at baseline should score well in the questionnaire, and patients who have a poor clinical status at baseline should score poorly
Responsiveness	Responsiveness refers to the ability of a measure to reflect underlying change. Preliminary responsiveness of the EAPIQ was assessed by comparing EAPIQ scores in those patients who report a change in their health status over the two-week period. Patients who were assessed at baseline and two weeks later were stratified by their report of worsening, no change and improvement in their 'overall health', 'all allergies', and 'eye allergy' symptoms, over the 7 to 10 days

Known-group validity was assessed by comparing EAPIQ scale scores between patients grouped according to their self-rating of ocular allergy severity. These patient-rated severity subgroups were compared using analysis of variance (ANOVA) on baseline data. It was hypothesised that patients in more severe groups would have worse (higher) EAPIQ scores, with the exception of the Treatment Satisfaction Scale.

Clinical validity was assessed by comparing EAPIQ scores according to the clinician report of ocular allergy severity. Severity was assessed using a single item measure that asks clinicians to rate the patient's ocular allergy as non-symptomatic, mild, moderate, or severe. Scores for non-symptomatic, mild, moderate and severe groups were expected to differ significantly from one another when compared using the Analysis of Variance (ANOVA) test.

In a preliminary analysis of responsiveness, changes in EAPIQ scores between baseline (week 0) and follow up (7–10 days after baseline) were compared among groups of patients who rated themselves as 'better', 'stable' or 'worsened' in terms of 'eye allergies', 'all allergies', and 'overall health' (as assessed using the Health Change Questionnaire). As a disease specific measure of allergy symptoms and wellbeing, EAPIQ scores were expected to be most sensitive to changes in 'eye allergies', and least sensitive to changes in 'overall health'.

Changes in EAPIQ scores were defined as small, moderate, or large using effect sizes (ES), as defined by Kazis [15]. Kazis proposed that an effect size between 0.20 and 0.49 are considered small, 0.50 to 0.79 are moderate, and 0.80 or above are large. It was hypothesised that those participants who reported an improved or worsened health status over the two weeks would show corresponding changes in EAPIQ scores, and those who reported an unchanged health status would have no significant change in their EAPIQ scores.

Statistical Analysis Software (SAS Institute Inc., Cary, NC) was used in the factor analysis assessment, clinical and known-group validity, and responsiveness over time. Multi-trait Analysis Program-Revised (MAP-R) [16] software was used for the assessment of psychometrics (internal consistency reliability, item convergent/divergent validity, floor/ceiling effects, scale/scale correlations). A significance level of 0.05 was used for all tests unless otherwise stated.

## Results

One hundred and forty six patients were recruited. Demographic and clinical characteristics for the patient population at baseline are presented in Table 2.

**Table 2:** Demographic and clinical characteristics

**Characteristic**	**n (%) or mean**
**Gender n (%)**	
Male	34 (23.29)
Female	99 (67.81)
*Missing data*	13 (8.90)
**Age**	
Mean	41.4
Standard deviation	13.3
Range	18.0–76.0
*Missing data*	1
**Ethnicity n (%)**	
Caucasian	114 (81.43)
African-American	2 (1.43)
Hispanic/Spanish American	10 (7.14)
Asian/Oriental/Pacific is.	6 (4.29)
Other	8 (5.71)
*Missing data*	6 (4.11)
**Highest level of education n (%)**	
High school or less	5 (3.62)
High school diploma	22 (15.94)
Some college	0 (0.00)
College degree	32 (23.19)
Graduate/postgraduate	47 (34.06)
Other	32 (23.19)
*Missing Data*	8 (5.48)
**Current work status n (%)**	
Working (FT/PT)	102 (71.33)
Retired – ill health	4 (2.80)
Retired – age	6 (4.20)
Never in paid employment	2 (1.40)
Unemployed/searching	12 (8.39)
Other	17 (11.89)
*Missing Data*	3 (2.05)
**Domestic situation n (%)**	
Living alone	15 (14.56)
Living with husband/partner	51 (49.51)
Living with children	9 (8.74)
Living with family/friends	25 (24.27)
Other	3 (2.91)
*Missing Data*	43 (29.45)
**Patient perceived severity of ocular allergy n (%)**	
I don't have eye allergy symptoms	26 (17.81)
Very mild	10 (6.85)
Mild	34 (23.29)
Moderate	42 (28.77)
Severe	26 (17.82)
Very Severe	7 (4.79)
*Missing data*	1 (0.68)
**Currently taking dry eye medication n (%)**	
Yes	93 (63.40)
No	52 (35.62)
*Missing data*	1 (0.68)

### Construct validity

Fourteen items in the EAPIQ asked for the percentage of time the patients were troubled while carrying out daily activities. Responses for these items were often missing or were inconsistent with responses for corresponding 'level of bother' items. Consequently, the 14 frequency of bother items (the second part of questions 18 to 31) were deleted. Principal Components Analysis (PCA) was then conducted on the remaining items using Varimax, Promax, and Oblimin rotation methods. Items 11 and 12 ('Please rate to what extent you usually suffer from eye allergy symptoms in relation to OVERALL allergy symptoms' and 'How many days in the past week have you been free from allergy symptoms', respectively) were deleted because they did not load on any of the factors.

In addition, items 23 'Trouble with putting on/wearing make-up' and 31 'Troubled by feeling uncomfortable in business settings' were excluded from further analyses because of the high frequency of missing data for these items. The high frequency of missing data for these items is likely due to a large number of patients (for example, men) who do not wear makeup or who do not work. As these two items provided important information about patients for whom there is relevance, the items have been retained as single items instead of being part of any scale scores.

The relative merits of assessing symptom-bother in a scale separate from symptom-frequency were assessed. Each of the symptom-bother items was highly correlated to its corresponding symptom-frequency item (range: r = 0.85–0.90), suggesting redundancy. Furthermore, when MAP-R analysis was performed with the symptom-frequency and symptom-bother items as two separate scales, the two scales correlated very highly (r = 0.90) with each other, again suggesting redundancy. Known-group validity testing suggested the superior discriminative ability of the symptom-frequency scale (F = 44.63 vs. 39.63). However, when symptom-bother and -frequency items were summed, discriminative validity was superior for the summed measure (F = 45.29). As a result, symptom-bother and symptom-frequency items were summed in the scoring, reducing 10 items to five in the final factor analysis and psychometric analyses.

To summarise, 16 items were dropped from the questionnaire (items 12, 13, and the second part of questions 18–31), two items were excluded from further analyses but retained as single item measures (items 23 and 31), and five symptom-frequency items (items 1–5)_were combined with five symptom-bother items (items 1–6) in the scoring. Thus 20 items were included in the final factor analysis. The final factor analysis resulted in four domains being established: Daily Life Impact (eight items), Psychosocial Impact (four items), Symptoms (five items) and Treatment Satisfaction (three items). Standardised regression coefficients are presented in Table 3. There were five items that loaded on more than one factor. These items were assigned to scales based on a qualitative assessment of their content (face validity).

**Table 3:** Final rotated factor pattern, Oblimin rotation method (Standardized Regression Coefficients).

		**Factor 1: Daily Life Impact**	**Factor 2: Psychosocial Impact**	**Factor 3: Symptoms**	**Factor 4: Treatment Satisfaction**
**22**	Troubled with concentrating on daily tasks	**0.89986**	-0.12534	0.01571	0.13486
**26**	Troubled by feeling irritable	**0.82799**	0.27741	-0.19979	-0.15545
**25**	Troubled by feeling frustrated/angry	**0.62794**	0.45009	-0.08244	0.04161
**24**	Troubled by feeling tired/fatigued	**0.61900**	0.01189	0.22953	0.01391
**21**	Troubled with sleeping	**0.58228**	0.19613	0.06456	-0.05398
**20**	Troubled with going outdoors	**0.55971**	0.17176	0.19318	0.08847
**18**	Troubled with reading	**0.53845**	-0.07626	0.47037	0.09245
**19**	Troubled with driving	**0.50325**	-0.03648	0.45243	0.05192
**29**	Troubled by feeling less attractive	-0.16437	**0.82591**	0.26851	0.06665
**30**	Troubled by feeling uncomfortable in social settings	0.15673	**0.80984**	-0.00459	-0.12471
**28**	Troubled by feeling helpless	0.19658	**0.70135**	-0.08085	0.12504
**27**	Troubled by feeling embarrassed	0.00565	**0.69426**	0.22569	0.12197
**3**	Red eyes	-0.09104	0.14292	**0.80942**	0.08124
**2**	Water eyes	0.10791	0.27184	**0.56104**	-0.13643
**1**	Swollen / puffy	0.01604	0.36873	**0.54369**	-0.10554
**5**	Dry eyes	0.49205	-0.24760	**0.51892**	-0.12212
**4**	Itchy / burning eyes	0.46646	0.10921	**0.45380**	0.00162
**32**	Satisfaction with eye drops	0.01473	-0.00548	-0.02048	**0.93682**
**34**	Satisfaction with comfort of eye drops	0.00802	0.01457	-0.02372	**0.93502**
**33**	Satisfaction with how quickly eye drops improved	0.00569	0.04313	-0.02938	**0.90542**

Results of tests of item convergent validity, item discriminant validity, reliability, and floor and ceiling effects are presented in Table 4. All items met the criterion for item convergent validity (item-scale correlations of ≥ 0.40), and 90.7% of item-scale correlations (adjusted for overlap) were higher with the item's own scale than with any other EAPIQ scale (criterion for item discriminant validity). Only three items (items 5 'Dry eyes', 24 'Troubled by feeling tired/fatigued', and 26 'Troubled by feeling irritable') correlated slightly higher with a scale other than their own, as compared to the correlation with their own scale.

**Table 4:** Results of tests of item convergent validity, item discriminant validity, reliability, and floor and ceiling effects for the EAPIQ (total sample)

		Item-level			Scale-level Reliability		Scale-level	
		
EAPIQ scale	No. of Items	Convergent validity^a^		Discriminant validity^b^	Internal consistency	Test-retest	Floor effects	Ceiling effects
		
		Range of correlations	Success rate (%)	Success rate (%)	Cronbach's alpha	ICC	%	%
Symptoms^c^	5	0.53–0.77	100	90	0.84	0.75	11.3	0.7
Daily Life Impact^c^	6	0.57–0.78	100	83.3	0.88	0.84	15.5	0.0
Psychosocial Impact^c^	6	0.58–0.75	100	91.7	0.88	0.85	28.2	0.0
Treatment Satisfaction^d^	3	0.84–0.86	100	100	0.93	0.72	1.7	0.0

^a ^Percentage of item-scale correlations ≥ 0.40.

^b ^Percentage of item-scale correlations (adjusted for overlap) higher with the item's own scale than with any other EAPIQ scale

^c ^Sample size of 142 patients who completed more than half of the items in the Daily Life Impact, Psychosocial Impact and Symptoms scales.

^d ^Sample size of 119 patients who completed more than half of the items in the Daily Life Impact, Psychosocial Impact and Symptoms and Satisfaction scales.

All scales demonstrated excellent internal consistency reliability, with alpha coefficients ranging from 0.84 to 0.93. In addition, all scales surpassed the 0.70 criterion for test-retest reliability [ICC coefficients ranged from 0.72 to 0.85]. These results demonstrate satisfactory reliability for the EAPIQ multi-item scales.

There were no significant ceiling effects (percentage scoring at ceiling ranged from 0% to 0.7%) for any of the EAPIQ scales when assessed for the total cross sectional sample. When floor effects were assessed in the total cross sectional sample there were significant floor effects (> 20%) for the Psychosocial Impact scale only (28.2% scoring at floor). Patients with 'no eye allergy symptoms', are expected to score at floor. When these patients were excluded, there were no significant floor effects (2.7% of scoring at floor for the Symptoms scale and 17.3% of scoring at floor for the Psychosocial Impact scale).

### Concurrent validity

EAPIQ Symptoms, Daily Life Impact, and Psychosocial Impact scores all correlated significantly with the miniRQLQ scores (P < 0.0001 for all). The correlations were moderate, ranging from r = 0.34 to r = 0.85. There was a low, statistically significant correlation between EAPIQ Treatment Satisfaction scores and miniRQLQ Eye Symptoms scores (r = 0.24, P = 0.0090). The EAPIQ Treatment Satisfaction Scale did not correlate significantly with any of the other miniRQLQ scales.

Correlations between the EAPIQ scales and the items of the HUI2/3 were low (0.20<r<0.45) or negligible and not statistically significant. These lower correlations were expected because the HUI2/3 is a generic health status measure, whereas the EAPIQ and the miniRQLQ are measures specific to ocular allergies.

### Comparison of EAPIQ scores according to patient demographics

**Figure 1:**
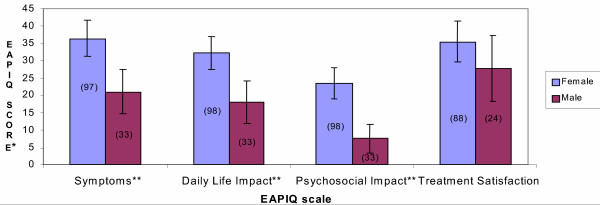
EAPIQ scale scores at baseline by gender. *Mean EAPIQ scores with 95% Confidence Interval (n) **Overall ANOVA results found statistically significant differences between groups (P < 0.01)

Scores from female subjects were significantly higher than those from male subjects for the EAPIQ Symptoms (F = 9.58, P = 0.0024), Daily Life Impact (F = 10.02, P = 0.0019), and Psychosocial Impact (F = 14.66, P = 0.0002) scales (Figure 1). Treatment Satisfaction scores did not differ by gender (F = 1.11, P = 0.2940).

**Figure 2:**
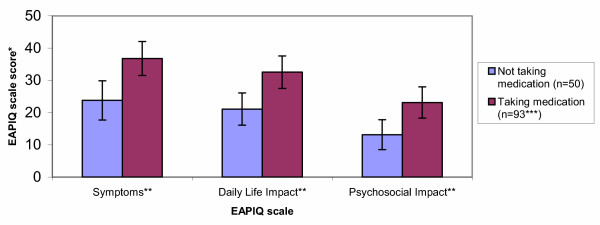
Comparison of EAPIQ scores at baseline between patients taking medication and those not taking medication for their eye allegy symptoms. *Mean EAPIQ scores with 95% Confidence Interval **Overall ANOVA results found statistically significant differences between groups (P < 0.01) ***Except for the Symptoms scale, for which n = 92

None of the EAPIQ scale scores correlated significantly with age, or with years of suffering from eye allergy symptoms. Patients taking medication for their eye allergy symptoms had significantly higher scores than those not taking medication for the EAPIQ scales of Symptoms (F = 9.10, P = 0.0030), Daily Life Impact (F = 8.31, P = 0.0046), and Psychosocial Impact (F = 6.92, P = 0.0095) (Figure 2). Treatment Satisfaction scores were not compared between these two groups, as individuals not on treatment did not complete the questions corresponding to the Treatment Satisfaction scores.

### Known-group validity

**Figure 3:**
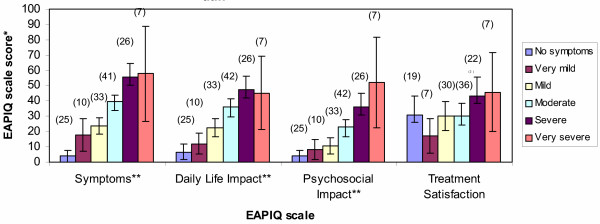
Known groups validity: EAPIQ scale scores at baseline by patient rating of ocular allergy severity. *Mean EAPIQ scores with 95% Confidence Interval (n) **Overall ANOVA results found statistically significant differences between groups (P < 0.0001)

Known-group validity estimates how well the questionnaire discriminates between groups. The results from the ANOVA test showed that the EAPIQ Symptoms (F = 27.96, P < 0.0001), Daily Life Impact (F = 16.88, P < 0.0001), and Psychosocial Impact (F = 14.97, P < 0.0001) scales distinguish between patients who rated themselves as having no allergy symptoms versus different grades of symptom severity (very mild, mild, moderate, severe, and very severe). (Figure 3).

EAPIQ Treatment Satisfaction scores did not differ significantly between groups of varying patient-rated severity. The result was expected since Treatment Satisfaction is not expected to change with severity.

### Clinical validity

**Figure 4:**
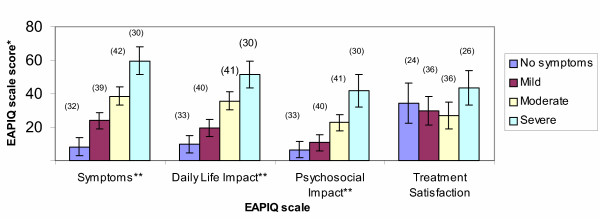
**Clinical validity: EAPIQ scale scores at baseline by clinician rating of ocular allergy severity**. *Mean EAPIQ scores with 95% Confidence Interval (n) **Overall ANOVA results found statistically significant differences between groups (P < 0.0001)

The patients' clinicians were asked to rate each patient as having either no eye allergy symptoms, mild eye allergy symptoms, moderate eye allergy symptoms or severe eye allergy symptoms. EAPIQ scale scores were compared among these four groups. Results from the ANOVA test showed that the EAPIQ scales of Symptoms (F = 46.95, P < 0.0001), Daily Life Impact (F = 34.55, P < 0.0001), and Psychosocial Impact (F = 24.83, P < 0.0001) distinguished with statistical significance between the patients in the no allergy symptoms category and the different severity groups as rated by clinicians (Figure 4). As expected, Treatment Satisfaction scores did not differ significantly between clinician-rated severity groups.

### Responsiveness

For comparisons among the 'better', 'stable' and 'worsened' groups according to all 3 health change items, small sample sizes (N<20) in the 'better' and 'worsened' groups warrant cautious interpretation.

**Figure 5:**
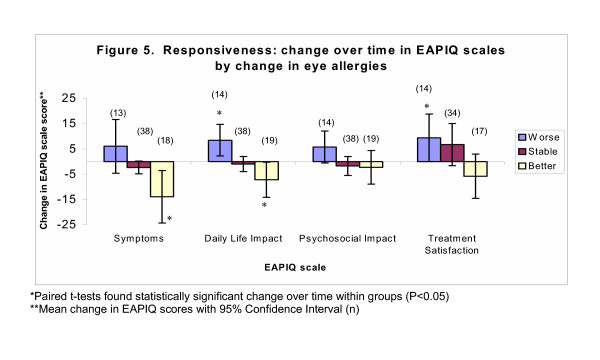
Responsiveness: change over time in EAPIQ scales by change in eye allergies. *Paired t-tests found statistically significant change over time within groups (P < 0.05) **Mean change in EAPIQ scores with 95% Confidence Interval (n)

The EAPIQ is responsive to changes in eye allergies. For all EAPIQ scales, scores worsened (ES range: 0.26 to 0.50) for patients who reported a deterioration of their eye allergy, improved (ES range: -0.10 to -0.56) for the group with 'better' eye allergies, and showed negligible or small change (ES range: -0.05 to 0.20) in patients who reported 'stable' eye allergies (Figure 5). However, sample sizes were not large enough to make statistical comparisons among the groups.

Findings were similar when comparing changes in EAPIQ scale scores according to change in 'all allergies', and 'overall health'.

## Discussion

Based on the results of this psychometric evaluation, the EAPIQ was found to be reliable, valid, and responsive. Following item reduction, scaling assumptions were met satisfactorily for the 4 multi-item scales and most items. The results provide evidence of the psychometric integrity of the EAPIQ within the studied eye allergy population and support its use in patients with eye allergies.

Findings suggest that asking patients to write in their responses can lead to inconsistent responses or missing data. Having items which required patients to rate the frequency with which they were bothered by their eye allergies while carrying out activities, in addition to their level of bother, proved confusing for some subjects. Furthermore the frequency of bother items which required patients to write in their responses also had high levels of missing data. Consequently, frequency items were deleted from the questionnaire and excluded from the remainder of the analyses.

High correlations between the symptoms 'intensity of bother' items (items 6–10) and their corresponding 'frequency' items (items 1–5) suggest redundancy. Clinicians view frequency as the more pertinent index of severity, whereas patients consider intensity of bother to be of greater salience. Consequently, instead of deleting either the 'intensity of bother' or 'frequency of bother' items, they were combined in the scoring. The 'intensity of bother' and 'frequency of bother' items were summed, not multiplied because known-group validity testing indicated superior discriminative ability for combining the items by summation rather than multiplication.

Items 23 and 31, pertaining to wearing makeup and working in business settings, respectively, were not included in the psychometric validation analyses due to high levels of missing data. However, these items provide important information for whom these items are relevant. Therefore, these items are scored as single items (not forming part of any scale score) rather than being excluded from the questionnaire entirely.

The final factor analysis suggested four domains: Daily Life Impact (eight items), Psychosocial Impact (four items), Symptoms (five items) and Treatment Satisfaction (three items), and two single items (wearing makeup and working in business settings). There were five items that loaded on two factors and these double loadings were logical in terms of content validity. For example, 'troubled with reading' item loaded on both the Daily Life Impact' factor (regression coefficient of 0.54), and the Symptoms factor (regression coefficient of 0.47). In terms of face validity, 'trouble with reading' is expected to be part of the Daily Life Impact factor. Since reading may be affected by the severity of eye allergy symptoms, it is logical that it also loads on the Symptoms factor.

The four multi-item scales of the EAPIQ were psychometrically robust; in that, all scales demonstrated excellent item convergent validity, excellent internal consistency reliability, and satisfactory test-retest reliability. All but three items satisfied the requirements for item discriminant validity. Floor and ceiling effects were satisfactory for the EAPIQ scales when patients with 'no current symptoms' who would be expected to score at floor were excluded from analysis.

Moderate correlations between the Symptoms, Daily Life Impact and Psychosocial Impact scales indicate that these three scales are measuring concepts that are related but distinguishable and not redundant. The low correlations between the Treatment Satisfaction scale and the remaining EAPIQ scales are also in line with expectations, as satisfaction as a concept is not expected to be strongly associated with symptom severity or impact on either the daily life or psychosocial factors.

When correlations were examined between the EAPIQ scales and the concurrent measures, correlations between similar scales were moderate or high, confirming the concurrent validity of the EAPIQ. Correlations between the concurrent measures and the treatment satisfaction scale were low, as expected since treatment satisfaction is not generally related to symptom severity or disease impact.

The EAPIQ scales correlated higher with the scales of the miniRQLQ than with the items of the HUI2/3. This finding was expected since the HUI2/3 is a generic health status measure, whereas the EAPIQ and the miniRQLQ are specific to ocular allergies. Thus, the EAPIQ demonstrated satisfactory concurrent validity.

The EAPIQ Symptoms, Daily Life Impact and Psychosocial Impact scales were able to distinguish between varying levels of patient-reported symptom severity. Higher scores (indicating worse health) were observed for more severe symptom severity, confirming the known-group validity of the EAPIQ. In line with expectations, Treatment Satisfaction scores did not change with varying degrees of patient-perceived symptom severity.

The EAPIQ Symptoms, Daily Life Impact, and Psychosocial Impact were also able to discriminate between varying levels of clinician-rated symptom severity, as evident by higher scale scores (indicating worse health) for patients with more severe symptom severity. Treatment Satisfaction scores did not change with varying degrees of clinician-reported symptom severity. These findings demonstrate the clinical validity of the EAPIQ.

Analyses of responsiveness were exploratory and should be interpreted with caution due to small sample sizes in the 'better' and 'worse' groups. For patients who reported their eye allergies as better, worsened, or stable between baseline and follow up, there were corresponding changes in scores for the scales of Symptoms, Daily Life Impact, Psychosocial Impact and Treatment Satisfaction. However, changes in EAPIQ scores in 'worsened' and 'better' groups were not consistently statistically significant.

Similar patterns were found for the change in EAPIQ scores according to 'change in overall heath' and 'change in eye allergies' even though the EAPIQ change scores for patients who rated their status as 'stable' were larger. These results suggest that the EAPIQ is responsive to change over time, but further study in a larger population is required to verify this assertion.

Minimal important differences or minimal important change over time were not examined for the EAPIQ scales. Knowledge of minimal important differences are important for interpreting the meaning of HRQoL results, thus, in future studies some attempt should be made to define minimal important differences for the EAPIQ.

## Conclusion

Results suggest that the EAPIQ is a reliable and valid measure and is appropriate for use in studies of ocular allergy. The EAPIQ is appropriate for assessing ocular allergy symptoms and their impact on patients' daily lives and psychosocial functioning, in addition to satisfaction with treatment. Further research investigating responsiveness to change over time and minimal important difference for the EAPIQ is warranted. Use of the EAPIQ in future research may contribute to a greater understanding of the impact of ocular allergies on patients' lives and ultimately lead to the use of treatments that improve functioning in the areas that are of greatest importance to the patients themselves.

## Authors' contributions

*PB, JW CB and LA conceived the study and participated in the design of the study. MA, WB, PB and CB coordinated the data collection. RA and LA oversaw the statistical analysis and drafted the manuscript. All authors were involved in item reduction decisions. All authors read and approved the final manuscript*.

## Supplementary Material

Additional File 1 Appendix: Eye Allergy Patient Impact Questionnaire Click here for file
