# Supplementary material for: The reliability, validity, and preliminary responsiveness of the Eye Allergy Patient Impact Questionnaire (EAPIQ)
Source: Health Qual Life Outcomes. 2005 Oct 31;3:67. doi: 10.1186/1477-7525-3-67 (PMC1291386; doi:10.1186/1477-7525-3-67)
Supplement: Additional File 1 — Appendix: Eye Allergy Patient Impact Questionnaire [file 1477-7525-3-67-S1.doc]

**APPENDIX**

**Eye Allergy Patient Impact Questionnaire**

The following questions refer to Seasonal Eye Allergy Symptoms you may have or have had. The time period in each question may be different, so please read each question carefully. Please answer each question by writing in a number or marking/circling the number which best describes your situation.

Your answers, combined with answers from other allergy sufferers, will help us determine the impact of **eye** allergy symptoms. **All answers will be kept confidential.** Please answer each question to the best of your ability. Answers should come from you alone, not family, friends, or office personnel.

Thank you for you time and participation.

# Section 1.

# Occurrence of Eye Allergy Symptoms

On a scale of 1 to 6, in the past week, how often did you **SUFFER** from each of the **eye** allergy symptoms below as they relate to either or both of your eyes (1 = none of the time, 6 = all of the time):

None of All of
 the time the time

1. Swollen/puffy eyes or eyelids: 1 2 3 4 5 6
2. Watery eyes: 1 2 3 4 5 6
3. Red eyes: 1 2 3 4 5 6
4. Itchy/burning eyes: 1 2 3 4 5 6
5. Dry eyes: 1 2 3 4 5 6

On a scale of 1 to 6, in the past week, how much were you **TROUBLED** by the following **eye** allergy symptoms: (1 = not troubled at all, 6 = extremely troubled)

Not troubled Extremely
 at all troubled

6. Swollen/puffy eyes or eyelids: 1 2 3 4 5 6

1. Watery eyes: 1 2 3 4 5 6
2. Red eyes: 1 2 3 4 5 6
3. Itchy/burning eyes: 1 2 3 4 5 6
4. Dry eyes: 1 2 3 4 5 6

# Section 2.

# Actions You May Have Taken For Your EYE Allergy Symptoms

13. How often have you visited each of the following types of **healthcare providers** for your **eye** allergy symptoms over the past year?

***Write in the number of visits in the past year.***

***If you did not visit a type of healthcare provider listed write in zero.***

**Number of Visits**

**TYPE OF HEALTHCARE PROVIDERS Related to Allergy Symptoms**

General/Practitioner/Internist

Ophthalmologist

Allergist

Optometrists/Opticians

Pharmacists/Chemists

Other: ____________________

# Section 3.

# Effect of EYE Allergy Symptoms on Everyday Activities and Emotions

14. In the past week, where did you spend the majority of your awake hours?

Indoors  Outdoors

15. Which of the following best describes your occupation:

Manual  Skilled  Managerial  Professional

16. In the past week, how many days have you performed tasks at work, school, and home **with eye** allergy symptoms,? (Answer may range from 0 to 7)

______ (# of days)

1. Generally, on the days you performed tasks at work, school, and home **with eye** allergy symptoms, how effective were you?
   For example if you answer 70% - you are indicating you performed at about 70% of your usual effectiveness level on the days you performed with **eye** allergy symptoms.
   (100% = your usual full-effectiveness level.)

_______ % Effectiveness at work with **eye** allergy symptoms

**In the past week:**

- On a scale of 1 to 6, how **TROUBLED** have you been with performing the following activities in the past week as a result of your **eye** allergy symptoms? (1 = not troubled at all, 6 = extremely troubled)

Not troubled Extremely
 at all troubled

18. Reading 1 2 3 4 5 6

19. Driving 1 2 3 4 5 6

20. Going outdoors 1 2 3 4 5 6

21. Sleeping 1 2 3 4 5 6

22. Concentrating on daily tasks 1 2 3 4 5 6

23. Putting on / wearing make-up 1 2 3 4 5 6

**In the past week:**

- On a scale of 1 to 6, how **TROUBLED** have you been by the following emotions in the past week as a result of your **eye** allergy symptoms? (1 = not troubled at all, 6 = extremely troubled)?

How Troubled?

Not troubled Extremely
 at all troubled

24. Feeling tired / fatigued 1 2 3 4 5 6

25. Feeling frustrated / angry 1 2 3 4 5 6

26. Feeling irritable 1 2 3 4 5 6

27. Feeling embarrassed 1 2 3 4 5 6

28. Feeling helpless 1 2 3 4 5 6

29. Feeling less attractive 1 2 3 4 5 6

30. Feeling uncomfortable
in social settings 1 2 3 4 5 6

31. Feeling uncomfortable
in business settings 1 2 3 4 5 6

# Section 4.

# Satisfaction With Treatment of Eye Allergy Symptoms

1. Please rate your overall level of satisfaction with your current eye drops for your **eye** allergy symptoms:

Very satisfied  Very Dissatisfied

Somewhat satisfied  Somewhat Dissatisfied

Satisfied  Dissatisfied

33. Please rate your overall level of satisfaction with how quickly your current eye drops improved your **eye** allergy symptoms?

Very satisfied  Very Dissatisfied

Somewhat satisfied  Somewhat Dissatisfied

Satisfied  Dissatisfied

34. Please rate your overall level of satisfaction with the overall comfort of your current eye drops for your **eye** allergy symptoms?

Very satisfied  Very Dissatisfied

Somewhat satisfied  Somewhat Dissatisfied

Satisfied  Dissatisfied

35. Did you try to do any activities this past week that you have avoided in the past because of your **eye** allergy symptoms?

NO

YES If yes, please list activities:_____________________
